# Renal ^99m^Tc-DMSA pharmacokinetics in pediatric patients

**DOI:** 10.1186/s40658-021-00401-7

**Published:** 2021-07-20

**Authors:** Donika Plyku, Michael Ghaly, Ye Li, Justin L. Brown, Shannon O’Reilly, Kitiwat Khamwan, Alison B. Goodkind, Briana Sexton-Stallone, Xinhua Cao, David Zurakowski, Frederic H. Fahey, S. Ted Treves, Wesley E. Bolch, Eric C. Frey, George Sgouros

**Affiliations:** 1 The Russell H. Morgan Department of Radiology and Radiological Science, Johns Hopkins University, School of Medicine, Baltimore, MD USA; 2 J. Crayton Pruitt Family Department of Biomedical Engineering, University of Florida, Gainesville, FL USA; 3 Department of Radiology, Faculty of Medicine, Chulalongkorn University and King Chulalongkorn Memorial Hospital, Thai Red Cross Society, Bangkok, Thailand; 4 Division of Nuclear Medicine and Molecular Imaging, Boston Children’s Hospital, Harvard Medical School, Boston, MA USA; 5 Departments of Anesthesiology and Surgery, Boston Children’s Hospital, Harvard Medical School, Boston, MA USA; 6 Division of Nuclear Medicine and Molecular imaging, Department of Radiology, Brigham and Women’s Hospital, Harvard Medical School, Boston, MA USA

**Keywords:** DMSA, Pediatric imaging, Compartmental modeling, Pharmacokinetics, Dose reduction/optimization

## Abstract

**Abstract:**

^99m^Tc-DMSA is one of the most commonly used pediatric nuclear medicine imaging agents. Nevertheless, there are no pharmacokinetic (PK) models for ^99m^Tc-DMSA in children, and currently available pediatric dose estimates for ^99m^Tc-DMSA use pediatric *S* values with PK data derived from adults. Furthermore, the adult PK data were collected in the mid-70’s using quantification techniques and instrumentation available at the time. Using pediatric imaging data for DMSA, we have obtained kinetic parameters for DMSA that differ from those applicable to adults.

**Methods:**

We obtained patient data from a retrospective re-evaluation of clinically collected pediatric SPECT images of ^99m^Tc-DMSA in 54 pediatric patients from Boston’s Children Hospital (BCH), ranging in age from 1 to 16 years old. These were supplemented by prospective data from twenty-three pediatric patients (age range: 4 months to 6 years old).

**Results:**

In pediatric patients, the plateau phase in fractional kidney uptake occurs at a fractional uptake value closer to 0.3 than the value of 0.5 reported by the International Commission on Radiological Protection (ICRP) for adult patients. This leads to a 27% lower time-integrated activity coefficient in pediatric patients than in adults. Over the age range examined, no age dependency in uptake fraction at the clinical imaging time was observed. Female pediatric patients had a 17% higher fractional kidney uptake at the clinical imaging time than males (*P* < 0.001).

**Conclusions:**

Pediatric ^99m^Tc-DMSA kinetics differ from those reported for adults and should be considered in pediatric patient dosimetry. Alternatively, the differences obtained in this study could reflect improved quantification methods and the need to re-examine DMSA kinetics in adults.

**Supplementary Information:**

The online version contains supplementary material available at 10.1186/s40658-021-00401-7.

## Introduction

The activity administered to pediatric nuclear medicine patients is currently based on the joint, consensus guidelines from the Society of Nuclear Medicine and Molecular Imaging (SNMMI) and the European Association of Nuclear Medicine (EANM) [1–3]. These guidelines assure consistency across different institutions while also promoting dose reduction; however, the recommended administered activities are based on a consensus approach rather than on a rigorous and quantitative approach. ^99m^Tc-dimercaptosuccinic acid (DMSA) is one of the most commonly used pediatric nuclear medicine imaging agents. Nevertheless, there are no pharmacokinetic (PK) models for ^99m^Tc-DMSA in children and currently available pediatric dose estimates for ^99m^Tc-DMSA use pediatric *S* values with PK data derived from adults using instrumentation and quantification techniques dating to the mid-70’s [4–9]. We have previously demonstrated that accounting for body habitus (height and weight) and organ size differences yields greater accuracy compared with a weight-only-based model [10–13]. As part of an ongoing dose optimization effort [10, 11, 14, 15], we examine whether the current reference (ICRP 53 [4]) DMSA PK model is consistent with model parameters obtained by ^99m^Tc-DMSA imaging quantification in children using current quantification techniques and imaging technologies.

## Materials and methods

### Patient data

**Fig. 1:**
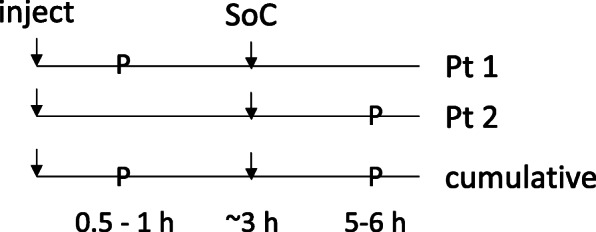
Imaging scheme. Standard of Care (SoC) imaging is combined with one other (protocol, p) imaging time point per patient after 2 patients we have 3 distinct imaging time points post-injection; one each at the protocol time point and two at the nominal 3 h standard of care imaging time

^99m^Tc-DMSA imaging data for pediatric patients were obtained from a combination of retrospective imaging data analysis and prospective data collection. Under an approved Institutional Review Board (IRB) protocol, data from 54 pediatric patients: 40 females and 14 males, ages ranging from 1 to 16 years old (Additional file 1) were retrospectively evaluated to extract the fraction of administered activity in the kidneys. These data were supplemented with prospective imaging in 23 (age range: 4 months to 6 years old) patients. To accommodate the special considerations in imaging pediatric patients prospectively, a data collection scheme was devised in which patients undergoing standard of care imaging were asked to consent to being imaged at one additional time point. No patient was asked to undergo more than one additional imaging time point (Fig. 1).

In the prospective study, two age groups were enrolled: less than 1 year, and between 4 to 6 years. In addition to the routine, standard-of-care, SPECT imaging, half of the subjects were imaged between 30 and 90 min, post-administration. The 2^nd^ half were imaged at 5–6 h, post-administration. Planar imaging was acquired at these non-standard-of-care time points and at the standard of care time point either immediately before or after the clinically indicated SPECT. In general, we did not find renal pathology (focal defects) to have a significant impact on overall kidney uptake; cases in which it did were excluded from our analysis.

### Activity quantification—retrospective study

Retrospective imaging analysis was performed by extracting single-photon emission computed tomography (SPECT) images from the Boston Children’s Hospital (BCH) image database. These were reconstructed using attenuation, scatter, and collimator response compensation.

**Fig. 2:**
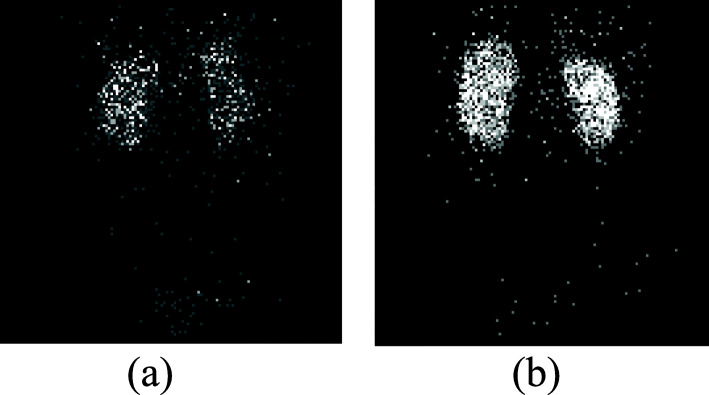
^99m^Tc-DMSA raw projection SPECT images of a representative patient **a** anterior and **b** posterior views

**Fig. 3:**
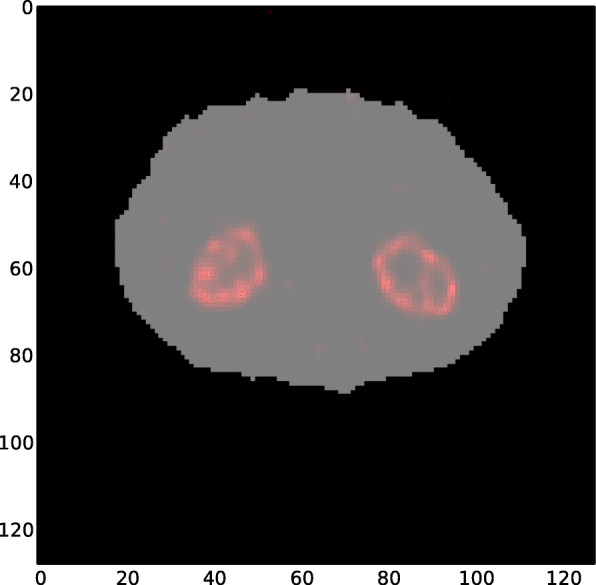
Attenuation map (shown in gray) and reconstructed SPECT image (kidney region displayed only, shown in red) of a representative patient, superimposed

DMSA projections were acquired on a Siemens ECAT or Symbia SPECT scanner using low-energy ultra-high resolution (LEUHR) parallel-hole collimators, a 15% energy window, 120 projection views for each detector over 360° using a body contouring orbit, and a duration of 8 s at each projection view. To enable attenuation compensation, we generated attenuation maps by reconstructing data from a scatter window (108–129 keV) using an initial attenuation map defined by the orbit of the camera [16]. The attenuation maps were then collapsed axially over a slice range spanning the kidneys. Thresholding was then used to define the body contour. The body contours generated in this manner were verified as being visually reasonable. The voxels inside the body contour were set to the attenuation coefficient of soft tissue at the 140 keV gamma energy of ^99m^Tc, repeated axially to span the kidney region, and used in the reconstruction. The activity distributions were reconstructed using the ordered subset expectation maximization (OS-EM) [17] algorithm with compensation for attenuation, the distance-dependent geometric collimator-detector response function, and scatter [18–20]. A total of 5 iterations with 16 subsets per iteration were used. Fig. 2a and b show the raw projection data for one of the patients, anterior and posterior views, respectively. Figure 3 shows the generated attenuation map and the reconstructed SPECT image (superimposed) for a representative patient SPECT study. A threshold was applied to the reconstructed images to define the kidneys and to obtain the sum of the reconstructed voxel value within the kidneys. This value was converted to activity using the measured sensitivity of the collimator–detector system and the total duration of the acquisition.

The camera system’s sensitivity was calculated using a phantom prepared by filling a 40-ml culture flask with 39.96 MBq of ^99m^Tc and enough water to cover the 3.5 × 6.5 cm^2^ area of a flat cell culture medium flask but not enough to cause significant attenuation. The phantom was placed on the collimator surface and a 1-min image was acquired with a 15% energy window, similar to that used clinically. The acquisition was repeated for the second detector. The activity was decay corrected to the time of the acquisition and the sensitivity of each detector in counts per minute per MBq (CPM/MBq) was determined.

The percent of injected activity (%IA) presented in the kidneys at the imaging time was calculated from the quantified data. This retrospectively collected patient data set provided fractional activity uptake in the kidneys at the imaging time, approximately 3 h post-injection. Since the field of view (FOV) in the provided SPECT images was limited to the abdominal area displaying the kidneys and since the uptake in the nearest organs of interest (liver and spleen) was low and at background level, it was not possible to quantify and extract activity uptake in the liver and spleen from this data set. Therefore, this data set provided a single activity time point for the kidneys only.

### Activity quantification—prospective study

For the 23 patients enrolled prospectively, renal SPECT was performed at 2–3 h post-injection (p.i.) of ^99m^Tc-DMSA. A planar image was also acquired at the clinical time point for all patients immediately after the SPECT acquisition. A 2^nd^ planar image was acquired between 15 and 90 min p.i. (13 patients, early group), or at 4–6 h p.i. (10 patients, delayed imaging group). SPECT images were reconstructed iteratively with attenuation, scatter, and collimator–detector response compensations. As CT images were not available, an attenuation map was generated from the reconstructed scatter window data. The renal activity was measured in the SPECT images and the %IA was calculated. Renal activity was also measured in both planar images and corrected for scatter, background and attenuation. Kidney activity from SPECT was used in conjunction with the planar image at the clinical time point to provide a factor to quantify the activity in the 2^nd^ planar image. The extracted PK data were used in the PK model development and validation.

### Mathematical fitting

Although the ICRP PK model as well as several authors have provided fitted expression for ^99m^Tc-DMSA PK for organs other than the kidneys, we found the uptake in these other organs to be too low for reliable quantification. Instead, we have fitted the general kinetic expression specified by the ICRP [4] to the kidney time–activity data obtained from pediatric patients, defined as:

1 $$ \frac{A_s(t)}{A_0}={F}_s{\sum}_i{a}_i\cdotp \exp\;\left(\frac{-\ln (2)}{T_i}\cdotp t\right); $$

where,

*A*_*s*_(*t*) — source (*S*) tissue activity as a function of time,

*A*_0_ — administered activity,

*F*_*S*_ — fraction of *A*_0_ in *S* at back-extrapolated time zero,

*a*_*i*_ — fraction of *F*_*S*_ obeying exponential kinetics with *T*_*i*_, and

*T*_*i*_ — half-life for exponential kinetic component, *i*.

Equation 1 gives the fraction of injected activity in an organ at each point in time; the equation assumes that there is immediate uptake in the organ (i.e. parameters are estimated assuming no uptake phase). The parameter, *F*_*S*_, as defined by the IRCP, in publication 53 is the fractional distribution to organ or tissue S (i.e. the fraction of the administered activity that would arrive in source organ or tissue S overall time if there were no radioactive decay); *a*_*i*_ is the fraction of *F*_*S*_ eliminated with a biological half-time *T*_*i*_.

The SAAM II software package (The Epsilon Group, Charlottesville, VA, USA) was used to obtain parameters values and their standard deviation by fitting Eq. 1 to the data [21]. The data were binned into 10-min increments for fitting (Table 1).

**Table 1:** Binned kidney activity fraction data used to fit expression 1 (from retrospective data analysis)

Time interval (h)	No. of patients	Time (h)	Average kidney activity fraction	St. dev. in kidney act. fraction	Coeff. of variation in kidney act. fraction (%)
0.17–0.32	1	0.24	0.096	-	-
0.50–0.65	3	0.58	0.167	0.014	8.4
0.67–0.82	4	0.74	0.215	0.063	29.2
0.83–0.98	1	0.91	0.300	-	-
1.17–1.32	2	1.24	0.193	0.071	37.1
1.67–1.82	1	1.74	0.434	-	-
1.83–1.98	1	1.91	0.321	-	-
2.17–2.32	2	2.24	0.283	0.002	0.8
2.33–2.48	2	2.41	0.317	0.027	8.5
2.50–2.65	9	2.58	0.335	0.028	8.4
2.67–2.82	14	2.74	0.320	0.066	20.7
2.83–2.98	17	2.91	0.294	0.065	22.3
3.00–3.15	22	3.08	0.326	0.065	20.0
3.17–3.32	5	3.24	0.283	0.086	30.3
3.33–3.48	2	3.41	0.336	0.046	13.7
3.50–3.65	1	3.58	0.353	-	-
3.67–3.82	1	3.74	0.244	-	-
3.83–3.98	1	3.91	0.433	-	-
4.00–4.15	1	4.08	0.341	-	-
4.17–4.32	3	4.24	0.366	0.058	15.7
4.33–4.48	1	4.41	0.344	-	-
4.67–4.82	2	4.74	0.395	-	-
5.67–5.82	1	5.74	0.474	-	-

### Statistical evaluation of age, weight, and sex differences

Quantile regression analysis on the 50th percentile (median regression) was used to assess the independent association between DMSA kidney activity fraction and age, weight, and sex. Median regression was used to account for potential non-normality of the outcome. Univariate and multivariable adjusted median regression analyses were performed with results presented as coefficients with corresponding 95% confidence intervals and *P* values. Statistical analysis was performed using Stata (version 16.0, StataCorp LLC, College Station, TX, USA).

## Results

**Fig. 4:**
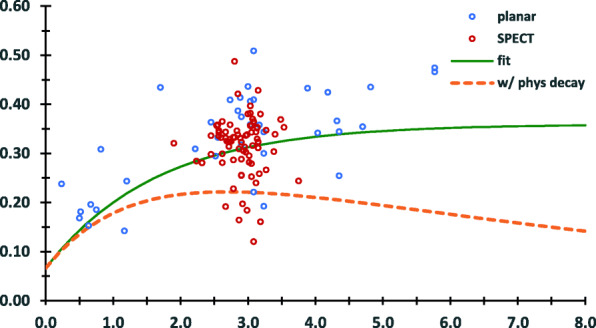
Fractional activity in kidneys as a function of time post-injection. The red circles, corresponding to data obtained from SPECT represent the clinical imaging time which is spread over time based on clinical logistics. The blue circles correspond to data obtained from planar imaging. The green solid line is the curve obtained from a fit to biological kinetics of DMSA (i.e. decay-corrected to the time of injection); the dotted orange curve corresponds to the actual imaging measurements obtained and reflect both the biological kinetics and physical decay of ^99m^Tc

The fractional kidney activity, calculated as the total activity measured in the kidneys divided by injected activity for the combined retrospective and prospective patients, was plotted according to the elapsed time between injection and imaging (Fig. 4).

Across the entire data set, the elapsed time ranged from 0.17 to 5.82 h. The clinically indicated SPECT was acquired as early as 1.9 to as late as 3.75 h after administration. A fit of Eq. 1 to the data shown on Fig. 4 gave an *F*_*S*_ = 0.3 ± 0.04, *a*_1_ = − 1.0, *T*_1_ = 1.1 ± 0.4, *a*_2_= 1.2 ± 0.9, *T*_2_ → ∞; a unique fit to the data was obtained only after *a*_1_ was fixed to − 1.0 and *T*_2_ was set to a very large number relative to the time scale of the data. Rearranging Eq. 1 using these parameters, the following equation is obtained:

2 $$ \frac{A_s(t)}{A_0}=0.3\left(1.2-\exp \left(\frac{-\ln (2)}{1.1\ h}\bullet t\right)\right) $$

Multiplying the right-hand side of Eq. 2 by the physical decay term for ^99m^Tc (i.e. $\exp \left(\frac{-\ln (2)}{6.0\ h}\bullet t\right)$) and integrating from zero to infinity gives $\frac{{\overset{\sim }{A}}_s}{A_0}=2.7\pm 0.4\ \mathrm{h}$.

**Fig. 5:**
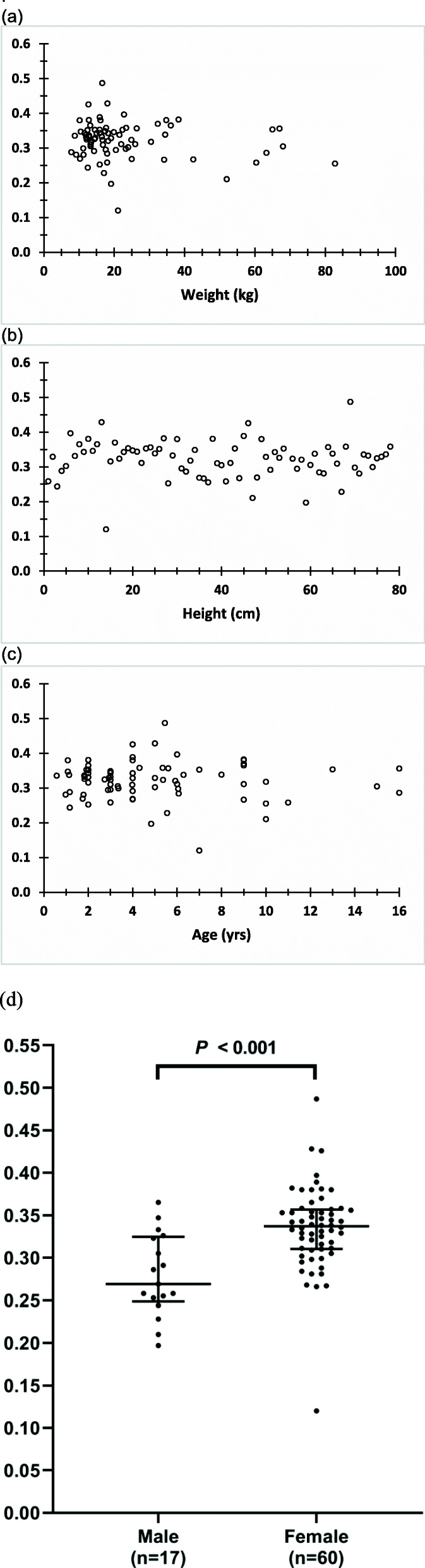
Kidney activity fraction at the clinical imaging time, approximately 3 h post DMSA administration. Data points shown are from quantitative SPECT imaging versus patient **a** weight, **b** height, **c** age and **d** gender (middle lines represent medians (50th percentile) and the interquartile ranges (25th–75th percentiles; top and bottom lines))

Figure 5 shows the renal activity fraction at the clinical imaging time as a function of patient weight, height, age, and sex.

The data set used in statistical analysis are summarized on Table 2. Tables 3 and 4 list the results of univariate and multivariate analysis, respectively. Weight and sex were found as significantly associated with kidney activity fraction. Using a multivariable median regression strategy, sex was an independent predictor of kidney activity fraction (coefficient for female sex (0.064) higher than males; 95% CI: 0.032–0.097; *P* < 0.001).

**Table 2:** Summary of data evaluated by regression analysis

Variable	***n*** (%) or median (interquartile range)
***N***	77
**Age (years)**	4 (2, 6)
**Age category**	
**0 to < 3 years**	24 (31.2%)
**3 to < 7 years**	36 (46.8%)
**7 to < 13 years**	13 (16.9%)
**≥ 13 years**	4 (5.2%)
**Weight (kg)**	17.4 (13.2, 24)
**Sex**	
**Male**	17 (22.1%)
**Female**	60 (77.9%)
**Kidney activity fraction**	0.33 (0.29, 0.35)

**Table 3:** Univariate median regression analysis of kidney activity fraction

Variable	Coefficient	95% CI	***P*** value
**Age (per year)**	− 0.002	(− 0.006, 0.002)	0.254
**Age category**			
**0 to < 3 years**	Reference	.	.
**3 to < 7 years**	− 0.004	(− 0.035, 0.027)	0.795
**7 to < 13 years**	− 0.014	(− 0.054, 0.026)	0.487
**≥ 13 years**	− 0.027	(− 0.09, 0.036)	0.393
**Weight (per 5 kg)**	− 0.004	(− 0.008, 0.001)	0.087
**Sex**			
**Male**	Reference	.	.
**Female**	0.067	(0.038, 0.096)	< 0.001*

Quantile regression on the median (median regression) was used to determine the univariate associations between each variable and kidney activity fraction.

*Statistically significant

**Table 4:** Multivariable median regression analysis of kidney activity fraction

Covariate	Adjusted Coefficient	95% CI	***P*** value
**Age (per year)**	0.003	(− 0.004, 0.011)	0.4
**Weight (per 5 kg)**	− 0.003	(− 0.012, 0.006)	0.532
**Sex**			
**Male**	Reference	.	.
**Female**	0.064	(0.032, 0.097)	< 0.001*

Multivariable quantile regression on the median (median regression) was used to determine the independent associations between each variable and kidney activity fraction.

*Statistically significant

## Discussion

The biokinetics of ^99m^Tc-DMSA are well documented for adult patients [4, 9] but not for pediatric patients. The age-specific absorbed and effective dose values listed in ICRP 128 [9] are adjusted for differences in anatomy but the biokinetic models used in these calculations are derived from adult data.

As part of an overall effort to revisit and further optimize the activity administered to pediatric patients for nuclear medicine imaging, we have recently examined the impact of a number of variables on image quality and the relationship between imaging quality, administered activity, pediatric patient absorbed dose and the risk of potential detrimental effects for ^99m^Tc-DMSA [10–15, 22–24].

In this work, we collected pharmacokinetic data for pediatric patients by combining quantitative SPECT and planar imaging. Planar imaging was quantified by calibrating the activity values obtained to those obtained from SPECT collected during the same imaging session (i.e. based on hybrid imaging technique [25]). Over the time points measured, we found that the count rate in the liver and spleen was too low to accurately assess kinetics in these tissues. We, therefore, focused on the kidneys and adopted the sum of exponential formulations used by the ICRP to parameterize the measurements. We also examined the fractional uptake at the clinical imaging time. Over the age range examined, we did not find a significant difference in the renal fractional uptake at the time of imaging, but we did find that the uptake in female patients was significantly higher than in male patients. We also found that a fit of the ICRP expression to our data gave different fitted parameter values. The plateau phase in fractional kidney uptake occurs at a fractional uptake value closer to 0.3 than the 0.5 reported by the ICRP. The data also suggest a longer time interval before the plateau is reached (solid green curve of Fig. 4). The dotted orange curve of Fig. 4 (which incorporates physical decay of ^99m^Tc) suggests that the clinical imaging time reflects an appropriate balance between biological uptake of the agent in the kidneys and physical decay of ^99m^Tc.

Although the imaging data were obtained from patients suspected of kidney pathology in the majority of cases, the overall kinetics were not affected by pathology. With accurate attenuation correction, SPECT quantification is typically within 5% [25]. Since we chose not to acquire CT to avoid unnecessary patient exposure, we used images generated from events collected in a scatter window to define the outer body contours for attenuation correction; this could introduce a 10-to-15% uncertainty. Based on this, we conclude that the 20-to-30% variation seen in kidney uptake at 3 h post DMSA administration (i.e. the “clinical” imaging time) most likely reflects patient-to-patient variability in DMSA uptake. Since the clinical diagnosis is primarily based upon identifying regions of diminished uptake, such overall variability does not impact the diagnostic imaging task but it does reflect that variability in kidney-absorbed dose for a pediatric patient population.

Evans et al. [26], presented ^99m^Tc-DMSA biokinetic data from children of different ages and degrees of renal dysfunction. Using planar imaging, Evans et al. obtained biokinetic information for liver and spleen where they measured maximum uptake values that were 10-fold less than found for the kidneys.

Table 5 compares the fitted parameters obtained in the current study with those reported by Evans et al. and those listed in ICRP publications 53 and 128. The last column of this table lists the time-integrated activity coefficient (TIAC), formerly called the residence time. The value obtained for pediatric patients obtained in this work is 27% lower than that reported by the ICRP; the value calculated from data reported by Evans is 19% lower but the standard deviation of the result is 100% of the value largely due to the very high standard deviation associated with *T*_2_.

**Table 5:** Comparison of Tc-99m DMSA model parameters (± standard deviations) for the kidneys

Parameter	*F*_*S*_	*T*_1_ (h)	*T*_2_ (h)	*a*_1_	*a*_2_	$$ \raisebox{1ex}{${\overset{\sim }{A}}_s$}\!\left/ \!\raisebox{-1ex}{${A}_0$}\right. $$ (h)
ICRP 53	0.5	1.0	∞	− 1	1	3.71
Evans et al.	0.4 ± 0.05	1.0 ± 0.2	7 ± 6	− 1	1	3 ± 3
Current study	0.3 ± 0.04	1.1 ± 0.4	∞	− 1	1.2 ± 0.9	2.7 ± 0.4

Longitudinal imaging data in pediatric patients for imaging agents including ^99m^Tc-DMSA have been severely lacking. The results presented herein suggest that the pediatric TIAC to kidney is lower than that reported by the ICRP and that, therefore, the absorbed dose and effective doses may also be lower. Over the age range examined, no age dependency in kidney fractional uptake was observed. A significantly (*P* < 0.001) higher fractional kidney uptake in female (0.33 ± 0.05) relative to male (0.38 ± 0.05) patients was also observed (Tables 3 and 4 and Fig. 5d). Correspondingly, the absorbed and the effective doses are expected to be higher for female pediatric patients than for males. Renal maturation appears to occur within the first year ([27, 28] Chapter 12). The data used for the ICRP DMSA model were collected in the mid-1970’s. Since no age dependency in uptake fraction at the time of imaging was observed, the difference between kinetic parameter values obtained in the current study and those reported in ICRP 53 and 128 may reflect an improvement in imaging quantification rather than a difference between pediatric patients and adults. A re-examination of adult DMSA kinetics may, therefore, be merited.

## Conclusions

Currently available pediatric dose estimates for ^99m^Tc-DMSA use pediatric *S* values with PK data derived from adults. We have examined whether the current reference (ICRP 53 [4]) DMSA PK model is consistent with model parameters obtained by ^99m^Tc-DMSA imaging measurements in children. Pharmacokinetics obtained in this study yield a 27% lower time-integrated activity coefficient in pediatric patients than in adults. Female pediatric patients had a 17% higher fractional kidney uptake at the clinical imaging time than males. These results suggest that a separate pediatric DMSA model is necessary to properly account for DMSA PK differences between children and adults. Furthermore, since adult DMSA kinetics were collected in the mid-70’s, a re-examination of adult DMSA kinetics is merited.

## Supplementary Information

**Additional file 1: Table 1.** Patients’ Characteristics Data.

## Data Availability

Data and materials related to this manuscript will be made available upon request.
