# Supplementary material for: Renal 99mTc-DMSA pharmacokinetics in pediatric patients
Source: EJNMMI Phys. 2021 Jul 20;8:53. doi: 10.1186/s40658-021-00401-7 (PMC8292521; doi:10.1186/s40658-021-00401-7)
Supplement: Supplementary file 1 — Additional file 1: Table 1. Patients’ Characteristics Data. [file 40658_2021_401_MOESM1_ESM.docx]

**Additional file 1**

Table 1. Patients’ Characteristics Data

| **Patient** | **Sex** | **Age (years)** | **Weight (kg)** | **Height (cm)** | **Injected activity kBq (mCi)** |
| --- | --- | --- | --- | --- | --- |
| 1 | M | 3 | 18 | 100 | 31.1 (1.15) |
| 2 | F | 5 | 12.1 | 109 | 19.8 (0.73) |
| 3 | M | 1.2 | 12.5 | 81 | 20.7 (0.76) |
| 4 | F | 1.2 | 7.82 | - | 11.9 (0.44) |
| 5 | F | 5 | 24 | 115.4 | 34.4 (1.27) |
| 6 | F | 6 | 22.8 | - | 32.8 (1.21) |
| 7 | F | 2 | 12.055 | 90 | 17.8 (0.66) |
| 8 | M | 9 | 36.1 | 136.4 | 52.8 (1.95) |
| 9 | F | 2 | 11.6 | 94 | 16.8 (0.62) |
| 10 | F | 9 | 34.8 | 138 | 50.4 (1.86) |
| 11 | F | 3 | 19.9 | - | 30.8 (1.14) |
| 12 | F | 2 | 13.2 | 88 | 21.4 (0.79) |
| 13 | F | 7 | 21.1 | - | 26.4 (0.98) |
| 14 | F | 5 | 18.1 | 109 | 31.7 (1.17) |
| 15 | F | 2 | 13.4 | 95.3 | 20.1 (0.74) |
| 16 | F | 9 | 32.4 | 142.5 | 47.1 (1.74) |
| 17 | M | 3 | 16.7 | 102.2 | 24.1 (0.89) |
| 18 | F | 4 | 18.3 | 101 | 27.1 (1.00) |
| 19 | F | 13 | 65.0 | 157.5 | 87.8 (3.25) |
| 20 | M | 1.1 | 10.5 | 77.50 | 15.4 (0.57) |
| 21 | F | 3 | 15.9 | 97 | 21.9 (0.81) |
| 22 | F | 6 | 22 | 119.10 | 32.3 (1.20) |
| 23 | F | 2 | 15.9 | 85.5 | 22.5 (0.83) |
| 24 | F | 16 | 67 | 157 | 86.2 (3.19) |
| 25 | F | 8 | 34.5 | 134 | 48.4 (1.79) |
| 26 | F | 2 | 12.4 | 98 | 19.0 (0.70) |
| 27 | F | 9 | 38.3 | 136 | 52.6 (1.94) |
| 28 | M | 2 | 15.9 | 94 | 27.9 (1.03) |
| 29 | F | 3 | 16.5 | 100 | 24.3 (0.90) |
| 30 | F | 13 | 10.2 | 77 | 14.0 (0.52) |
| 31 | F | 3 | 17.6 | 104.7 | 25.8 (0.95) |
| 32 | M | 16 | 63.3 | 163.50 | 88.6 (3.28) |
| 33 | F | 10 | 30.5 | 128 | 45.7 (1.69) |
| 34 | F | 3 | 17.4 | 102.5 | 24.7 (0.92) |
| 35 | F | 4 | 25 | 108.5 | 34.5 (1.28) |
| 36 | F | 9 | 34.2 | 103 | 52.6 (1.95) |
| 37 | M | 10 | 82.8 | 162 | 80.9 (3.00) |
| 38 | F | 2 | 12.8 | 90.0 | 20.3 (0.75) |
| 39 | F | 3 | 13.3 | 94.8 | 17.6 (0.65) |
| 40 | M | 15 | 68 | 174 | 87.9 (3.25) |
| 41 | M | 11 | 60.4 | 143.5 | 84.1 (3.11) |
| 42 | F | 9 | 26 | - | 37.6 (1.39) |
| 43 | F | 7 | 22.4 | 121.20 | 32.1 (1.19) |
| 44 | F | 4 | 42.5 | 115 | 57.9 (2.14) |
| 45 | F | 4 | 15.9 | 93 | 23.2 (0.86) |
| 46 | F | 4 | 12.7 | 87.3 | 18.1 (0.67) |
| 47 | M | 10 | 52 | 144.1 | 75.5 (2.79) |
| 48 | M | 21 mos | 10.3 | 82 | 15.7 (0.58) |
| 49 | F | 4 | 16.1 |  | 22.5 (0.83) |
| 50 | F | 4 | 19.1 | 105.8 | 29.7 (1.10) |
| 51 | M | 4 | 14.3 | 101.6 | 20.6 (0.76) |
| 52 | F | 4 | 15 | 101 | 21.4 (0.79) |
| 53 | M | 22 mos | 14.4 | 92 | 21.8 (0.81) |
| 54 | F | 23 mos | 14.5 | 89 | 21.2 (0.78) |
| 55 | F | 5.37 | 24.9 | 120 | 32.4 (1.20) |
| 56 | M | 2.89 | 20.5 | 109 | 28.1 (1.04) |
| 57 | F | 5.92 | 18.3 | 112.5 | 27.8 (1.03) |
| 58 | M | 4.83 | 19.1 | 110 | 24.3 (0.90) |
| 59 | F | 3.33 | 13.3 | 90 | 15.7 (0.58) |
| 60 | F | 1.14 | 12 | 77 | 15.9 (0.59) |
| 61 | F | 6.07 | 18 | 112 | 23.8 (0.88) |
| 62 | F | 0.98 | 9.2 | 75.5 | 11.9 (0.44) |
| 63 | F | 5.6 | 26.4 | 123 | 35.1 (1.30) |
| 64 | F | 6.29 | 21.5 | 120 | 28.9 (1.07) |
| 65 | F | 3.98 | 16.8 | 100 | 23.2 (0.86) |
| 66 | M | 5.55 | 17.1 | 107 | 20.3 (0.75) |
| 67 | F | 4.31 | 17.7 | 108 | 24.1 (0.89) |
| 68 | F | 5.45 | 16.6 | 109 | 24.1 (0.89) |
| 69 | F | 6.05 | 23.3 | 115 | 30.0 (1.11) |
| 70 | F | 1.79 | 11.3 | 87 | 14.3 (0.53) |
| 71 | F | 0.58 | 8.8 | 64 | 13.0 (0.48) |
| 72 | F | 1.84 | 12.9 | 91 | 15.4 (0.57) |
| 73 | F | 3.36 | 11.2 | 87 | 14.6 (0.54) |
| 74 | F | 2.74 | 12.2 | 82 | 17.3 (0.64) |
| 75 | F | 2.93 | 14.6 | 97 | 20.8 (0.77) |
| 76 | F | 1.82 | 12.9 | 82 | 16.2 (0.60) |
| 77 | F | 5.35 | 23.4 | 116 | 33.5 (1.24) |
